# Thionine modulates tau phosphorylation in an Alzheimer’s disease cell culture model

**DOI:** 10.55730/1300-0152.2756

**Published:** 2025-06-10

**Authors:** Seda ÖNDER, Kevser BİBEROĞLU, Özden TACAL

**Affiliations:** Department of Biochemistry, School of Pharmacy, Hacettepe University, Ankara, Turkiye

**Keywords:** Thionine, phenothiazine compounds, Alzheimer’s disease, tau phosphorylation, neurofibrillary tangles

## Abstract

**Background/aim:**

Tau protein, which is crucial for sustaining the cytoskeletal network by assisting microtubule construction, contributes significantly to the pathophysiology of Alzheimer’s disease (AD). The hyperphosphorylation of tau causes it to detach from microtubules (MTs), leading to the formation of neurofibrillary tangles (NFTs) in neurons, which ultimately results in cell death. Thionine (TH), a cationic phenothiazine-structured compound, has been the topic of extensive research due to its interesting physicochemical properties. It is a common biological dye, especially useful in histology due to its strong affinity for biological membranes. Furthermore, TH serves as a photosensitizer in phototherapy. It has a phenothiazine pharmacophore, which makes it selective against microbial and tumor cells. Our prior studies demonstrated that TH inhibits human plasma butyrylcholinesterase (BChE) by acting as a nonlinear inhibitor and also affects amyloid precursor protein (APP) metabolism in PS70 cells. In the current research, we investigated whether TH modulates the phosphorylation of tau in N2a/APPSwe cells.

**Materials and methods:**

Using flow cytometry, we identified the dose range and treatment time of TH that did not affect the viability of N2a/APPSwe cells. The western blot method was used to investigate the effects of TH on total tau and four key tau phosphorylation sites.

**Results:**

The results indicated that TH reduces tau phosphorylation at residues Ser202/Thr205, Ser396, Ser396/Ser404, and Thr181, which contribute to NFT formation.

**Conclusion:**

When all these findings are evaluated together, TH may have a therapeutic potential against AD.

## 1. Introduction

Alzheimer’s disease (AD) is identified by two key neuropathological markers in the brain: (i) senile plaques and (ii) neurofibrillary tangles (NFTs). While senile plaques are the result of the conversion of amyloid-β (Aβ) peptide into extracellular amyloid deposits, NFTs are associated with hyperphosphorylated tau protein (Janeiro et al., 2021). Mounting evidence indicates that amyloid-β accumulation promotes hyperphosphorylation of tau, causing synaptic disruption, tau aggregation, and the formation of NFTs (He et al., 2018).

Tau has been identified as a tubulin-associated protein since its discovery, as reported by Weingarten et al. (1975), due to its role in stabilizing axonal microtubules in the brain; hence, it regulates axon development and transport (Weingarten et al., 1975; Chaudhary et al., 2018). The processes underlying tau-associated neurodegeneration continue to be poorly understood. Consequently, present antitau approaches primarily focus on the aggregation of tau, which is believed to be harmful to neurons. Tau regulation can be affected by posttranslational modifications, which encompass a variety of modification processes, such as phosphorylation/dephosphorylation, glycation, O-linked N-acetylglucosamine (O-GlcNAc) glycosylation, oxidation, acetylation, sumoylation, nitration, methylation, and ubiquitination (Mietelska-Porowska et al., 2014). The functional characteristics of tau are intimately associated with posttranslational modifications. Phosphorylation and dephosphorylation are key changes indicating impaired function and catabolism of tau; this process depends on the balance between the activities of tau kinase and phosphatase (Sabbagh and Dickey, 2016). Phosphorylated tau (p-tau) accumulation is associated with neurodegeneration, synaptic dysfunction, and the onset of dementia (Xia et al., 2021). In AD, tau hyperphosphorylation results in its separation from microtubules (MTs) and the formation of paired helical filaments, which are found in dystrophic neurites and NFTs (Spillantini and Goedert, 2013). Therefore, it is critical to target these modifications concurrently to avert tau aggregation and recover the protein’s normal function. Early treatment can effectively prevent the development of additional pathologies, as these events commence before the manifestation of symptoms (Xia et al., 2021).

**Figure 1:**
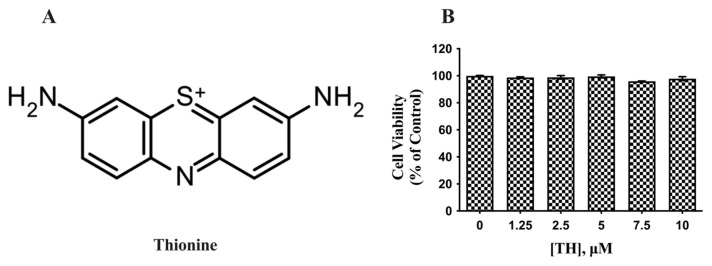
Effect of TH on the viability of N2a/APPSwe cells. (**A)** Chemical structure of TH. **(B)** After TH treatment, the viability of N2a/APPSwe cells was assessed by flow cytometric analysis (n = 3).

Phenothiazine compounds are used widely in various medical fields and research areas. They can efficiently block dopamine, serotonin, histamine, acetylcholine and α-adrenergic receptors (Darvesh et al., 2013). Although primarily used in the treatment of schizophrenia, phenothiazine compounds have been shown to have different biological roles, such as antibacterial, antiviral, antitumor, antihistamine and anthelmintic effects (Varga et al., 2017). This diversity suggests that the phenothiazine pharmacophore is a potential candidate for multitarget approaches (Sudeshna and Parimal, 2010). Thionine (hereafter referred to as TH), a planar tricyclic heteroaromatic molecule in the phenothiazine structure with a single positive charge, can bind to acidic proteins and nucleic acids (Figure 1A). In previous studies performed in our laboratory, the results showed that TH caused nonlinear inhibition of human butyrylcholinesterase (K_i_: 2.1 ± 0.42 μM) (Biberoglu et al., 2016). Additionally, TH was shown to reduce the secretion of Aβ40 and Aβ42 peptides in PS70 cells, a cellular model of amyloid pathology (Yuksel et al., 2017). Recently, we demonstrated that toluidine blue O (TBO), another phenothiazine compound structurally similar to TH, decreases both Aβ accumulation and tau phosphorylation (Onder et al., 2022). In our current research, we explored whether TH modulates tau phosphorylation at critical epitopes that contribute to the formation of NFTs. For this purpose, we used mouse neuroblastoma cells overexpressing the Swedish mutant of human APP695. Our findings revealed that TH decreases levels of total tau and p-tau at residues Ser202/Thr205, Ser396, Ser396/Ser404, and Thr181.

## 2. Materials and methods

### 2.1. Thionine and other reagents

Thionine acetate salt (TH; dye content approximately 90%, T3387) was purchased from Sigma-Aldrich (Sigma-Aldrich, St. Louis, MO, USA). BCA protein assay kit was purchased from Pierce (Thermo Fisher/Pierce, Rockford, IL, USA); Clarity ECL and ECL Max were from Bio-Rad (Bio-Rad, Hercules, CA, USA). Unless otherwise indicated, the other reagents were acquired from Thermo Fisher Scientific (USA) or Biological Industries (Israel).

### 2.2. N2a/APPSwe cell culture

N2a mouse neuroblastoma cells overexpressing the Swedish mutation (K595N/M596L) of human APP695 (referred to as N2a/APPSwe) were generously supplied by Prof. Gopal Thinakaran. In this research, the N2a/APPSwe cell line was preferred, as it is an important model for studying AD pathology. In this cell line, due to the specific mutation in APP, the cleavage of APP by β-secretase increases significantly, resulting in higher levels of Aβ peptides that are crucial in the pathogenesis of AD (Thinakaran et al., 1996). The N2a/APPswe cell line, which overexpresses the Swedish mutant of human APP, is widely used as an in vitro model for studying amyloidogenic processing. Importantly, increased Aβ levels in these cells have been reported to induce early tau hyperphosphorylation, partially modeling Aβ-driven tau pathology (Wang et al. 2006). N2a/APPSwe cells (750,000–1,000,000 cells/plate) were cultured in a medium comprising equal volumes of DMEM and Opti-MEM, enriched with 5% fetal bovine serum, 4 mM L-glutamine, 100 U/mL penicillin, 0.1 mg/mL streptomycin, and 0.2 mg/mL geneticin (G418 sulfate), in a CO_2_ incubator at 37 °C, with 5% CO_2_, as previously reported (Onder et al., 2022).

### 2.3. TH treatment and sample preparation

Fresh stock solutions of TH at concentrations between 0.5 and 4 mM were prepared in methanol and filtered through 0.2 μm membrane filters. After that, N2a/APPSwe cells were treated with 1.25–10 μM TH or 0.25% methanol-containing Opti-MEM medium (for control groups) for 24 h in a humidified atmosphere of a CO_2_ incubator. The final concentration of methanol in the cell culture medium was maintained at 0.25%. Following 24 h of treatment, the conditioned medium was removed, the cells were lysed using RIPA buffer mixed with a cocktail of phosphatase/protease inhibitors, and total protein content in the cell lysates was quantified using the BCA method, following the protocol reported in our previous studies (Yuksel et al., 2017; Onder et al., 2022).

### 2.4. Cell viability assay

Following TH treatment of N2a/APPSwe cells as indicated above, the culture medium was collected into fluorescence-activated cell sorting (FACS) tubes. The cells were rinsed with phosphate-buffered saline and dislodged by treatment with 0.25% trypsin-EDTA. Trypsin was neutralized with the collected culture medium, and the cells were collected and processed according to the protocol (Yuksel et al., 2017). Propidium iodide (PI) (1 μg/mL) was added to the cell suspensions for staining of dead cells, and stained cells were analyzed using FACSAria I flow cytometry (Biberoglu et al., 2019) (λ_excitation_ = 351 nm; λ_emission_ = 617 nm). Data from flow cytometry were acquired using BD Accuri C6 software (BD Biosciences, San Jose, CA, USA).

### 2.5. Immunoblotting

An equal volume of 2 × Laemmli buffer was mixed with N2a/APPSwe cell lysate, and the samples were denatured by boiling at 95 °C for 5–7 min. Equal amounts of protein per well were loaded into Any kD Mini-PROTEAN TGX Stain-Free precast gel (Bio-Rad, Hercules, CA, USA), separated by SDS-PAGE (20–40 μg protein/well, 200 V, approximately 40 min) (Yuksel et al., 2017), and transferred onto 0.45 μm polyvinylidene fluoride (PVDF) membranes. The primary antibodies used were as follows: [mouse anti-HT7 (1:1000; Thermo Fisher Scientific, Rockford, IL, USA); mouse anti-AT8 (1:200; Thermo Fisher Scientific, Rockford, IL, USA); mouse anti-AT270 (1:800; Thermo Fisher Scientific, Rockford, IL, USA); mouse anti-PHF-13 (1:200; Santa Cruz Biotechnology, Dallas, TX, USA); mouse anti-PHF-1 (1:100; generously supplied by Dr. Peter Davies); and mouse anti-β-actin (AC15, 1:10,000; Santa Cruz Biotechnology, Dallas, TX, USA)]. Blocking and washing processes were carried out according to the protocol defined in our previous studies (Yuksel et al., 2017; Onder et al., 2022). After that, the membranes were incubated with ECL/Clarity ECL Plus for 1–5 min to visualize protein bands. Images of membranes were captured by a Bio-Rad ChemiDoc XRS+ Imaging system (Bio-Rad, Hercules, CA, USA). ImageLab, version 6 software (Bio-Rad, Hercules, CA, USA), was used for densitometric analysis and normalization to β-actin (internal loading control) (Yuksel et al., 2017; Onder et al., 2022).

### 2.6. Data analysis

GraphPad Prism (version 10.3) software was used for statistical analyses of cell viability and Western blot data. Kruskal–Wallis nonparametric tests were used for comparison of data between all groups to identify doses that did not affect cell viability. Data collected from Western blot analyses were assessed using ordinary one-way ANOVA to identify differences among groups, followed by Dunnett’s post hoc test for pairwise comparisons relative to the control group. Data were presented as mean ± standard error of the mean (SEM). A p value less than 0.05 was considered statistically significant.

## 3. Results

### 3.1. Cell viability after TH treatment

We first assessed the cytotoxic effects of 1.25–10 μM TH on N2a/APPSwe cells by flow cytometry before analyzing its effects on tau. Based on flow cytometry results, no statistically significant difference was observed in the cell viability of N2a/APPSwe cells after 24 h.

### 3.2. Effect of TH on total tau

**Figure 2:**
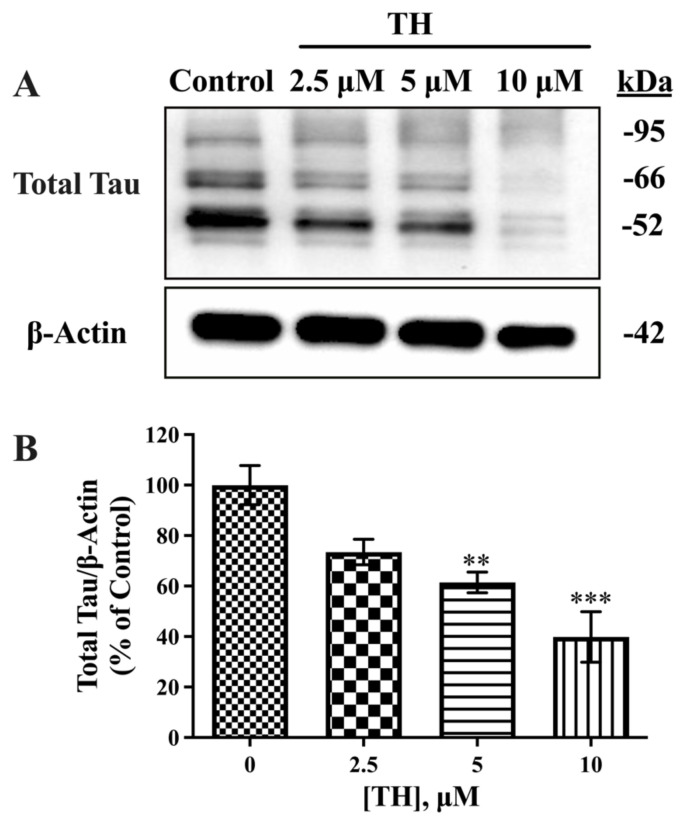
Effect of TH on total tau expression levels in N2a/APPSwe cells. **(A)** Immunoblot images of intracellular total tau and β-actin after TH treatment. **(B)** Densitometric analysis of intracellular total tau relative to β-actin (n = 3–5) (**, p < 0.01; ***, p < 0.001).

Next, we determined the effect of TH on total tau levels in N2a/APPSwe cells. Immunoblot analysis of total tau in cell lysates was performed using HT7 antibody. Multiple bands were observed in the Western blot images, with a range of approximately 50–100 kDa, as shown in Figure 2A. Densitometric analyses indicated that total tau expression levels in TH-treated cells significantly decreased after 24 h, and this reduction was dose-dependent. Significant decreases in total tau reached up to 39% and 60% for 5 μM and 10 μM TH, respectively, compared to the control group (Figure 2B).

### 3.3. Effect of TH on tau phosphorylation

**Figure 3:**
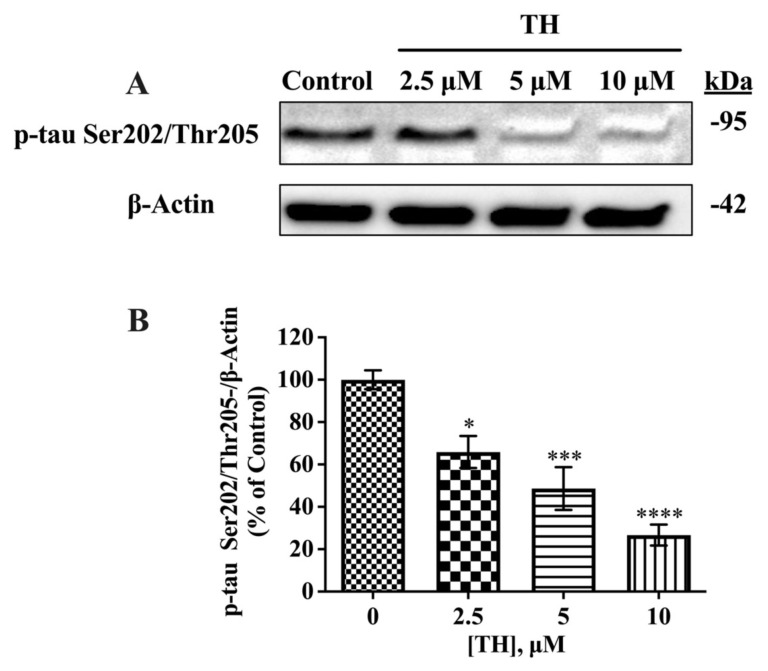
Effect of TH on p-tau Ser202/Thr205 expression levels in N2a/APPSwe cells. **(A)** Immunoblot images of intracellular p-tau Ser202/Thr205 and β-actin after TH treatment. **(B)** Densitometric analysis of p-tau Ser202/Thr205 relative to β-actin (n = 4) (*, p < 0.05; ***, p < 0.001; ****, p < 0.0001).

To determine whether TH affects tau phosphorylation, phosphorylation at unique tau epitopes was examined using AT8, AT270, PHF13, and PHF1 antibodies. The AT8 antibody identifies tau phosphorylated at Ser202/Thr205; the AT270 antibody is unique for p-tau at Thr181; the PHF13 monoclonal antibody specifically targets p-tau at Ser396, and the PHF1 monoclonal antibody detects p-tau at Ser396 and Ser404. Results from Western blots revealed that the expression levels of p-tau Ser202/Thr205 (approximately 89 kDa) were significantly decreased in a dose-dependent manner in TH-treated cells, relative to vehicle-treated cells. Significant reductions in p-tau Ser202/Thr205 expression levels were 34%, 51%, and 73%, respectively, for 2.5 μM, 5 μM, and 10 μM TH, as shown in Figures 3A and 3B.

**Figure 4:**
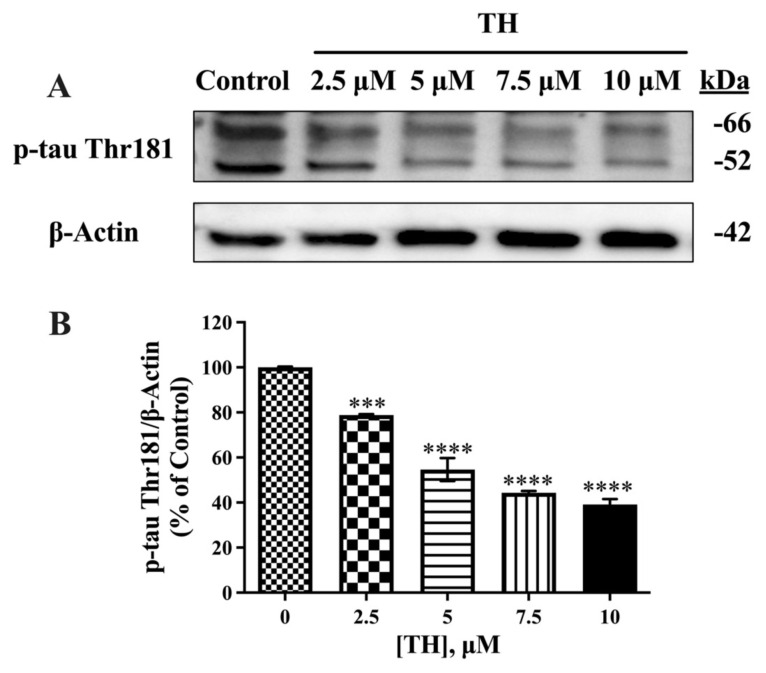
Effect of TH on p-tau Thr181 expression levels in N2a/APPSwe cells. **(A)** Immunoblot images of intracellular p-tau Thr181 and β-actin after TH treatment. **(B)** Densitometric analysis of p-tau Thr181 relative to β-actin (n = 3–4) (***, p < 0.001; ****, p < 0.0001).

The dominant bands of p-tau Thr181 were observed at 45 kDa and 64 kDa with the AT270 antibody (Figure 4A). Quantitative analysis revealed that the sum of the intensities of these two bands was significantly decreased in a dose-dependent manner compared to the control. The inhibitory effect of 2.5 μM, 5 μM, 7.5μM, and 10 μM TH on p-tau Thr181 expression levels was 21%, 33%, 55%, and 60%, respectively, compared to the control, as shown in Figure 4B.

**Figure 5:**
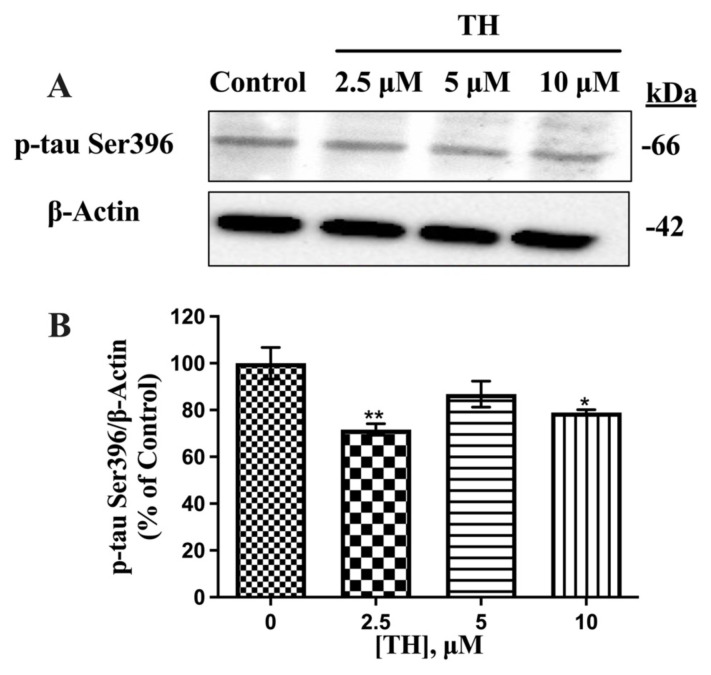
Effect of TH on p-tau Ser396 expression levels in N2a/APPSwe cells. **(A)** Immunoblot images of intracellular p-tau Ser396 and β-actin after TH treatment. **(B)** Densitometric analysis of p-tau Ser396 relative to β-actin (n = 3) (*, p < 0.05; **, p < 0.01).

Furthermore, PHF13 antibody-based Western blot results demonstrated that p-tau Ser396 (64 kDa) expression levels in N2a/APPSwe cells were significantly reduced by TH, as shown in Figures 5A and 5B. As a result of densitometric analysis, the significant decreases in p-tau Ser396 levels in the presence of 2.5 μM and 10 μM TH were 29% and 21%, respectively, compared to the control.

**Figure 6:**
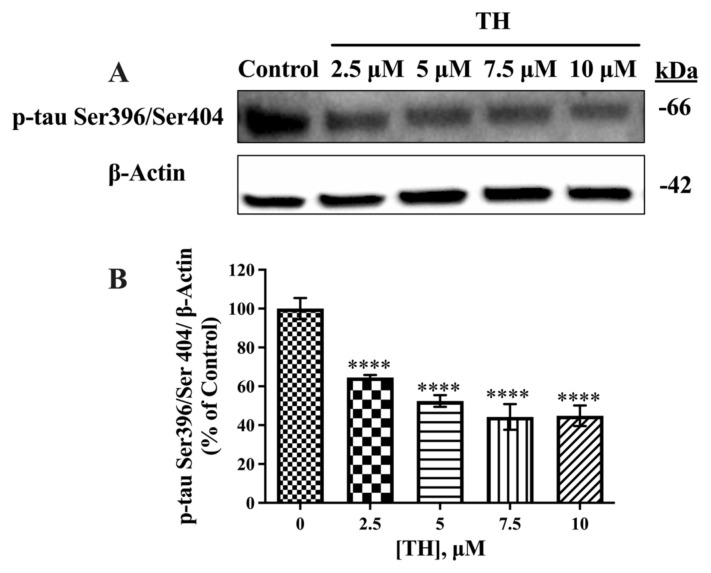
Effect of TH on p-tau Ser396/Ser404 expression levels in N2a/APPSwe cells. (**A)** Immunoblot images of intracellular p-tau Ser396/Ser404 and β-actin after TH treatment. **(B)** Densitometric analysis of p-tau Ser396/Ser404 relative to β-actin (n = 3–5) (****, p < 0.0001).

Densitometric analysis of Western blots revealed that the expression levels of p-tau Ser396/Ser404 (approximately 64 kDa) in cells treated with 2.5–10 μM TH were significantly decreased by 34%–55%, as shown in Figures 6A and 6B, compared to the control.

## 4. Discussion

The landscape of AD treatment is rapidly evolving, with significant advancements in understanding the disease’s complex pathophysiology and identifying novel drug targets. While current treatments primarily offer symptomatic relief, emerging therapies aim to modify the disease course by targeting underlying mechanisms such as tau pathology, amyloid accumulation, and neuroinflammation (Janeiro et al., 2021). The advancement of antitauopathy pharmaceuticals is a significant subject for investigation. The hyperphosphorylation of aggregated tau, the main component of AD-associated NFTs, is one of the earliest steps in the progression of AD, as revealed by its discovery (Sharma, 2019). The longest tau isoform expressed in the human central nervous system has over 85 phosphorylation sites, 80 serines/threonines, and five tyrosines. The phosphorylation pattern in AD evolves with disease progression. Previous research has demonstrated that tau’s capacity to bind to tubulin and facilitate MT formation can be altered by its phosphorylation. Early phosphorylation steps impair tau’s interaction with MT while promoting its relocalization to the somatodendritic compartment (Wang et al., 2013). Phosphorylation at multiple sites causes conformational changes that facilitate tau misfolding and aggregation (Alonso et al., 2001). Research conducted by Jeganathan et al. (2008) indicates that pseudophosphorylation of experimental tau promotes conformations that enhance tau aggregation by modifying the rigidity of the paperclip conformation. Pseudophosphorylation of tau at Ser199, Ser202, and Thr205 separates the N-terminal domain from the C-terminal domain, while pseudophosphorylation at Ser396 and Ser404 detaches the C-terminal domain from the repeat domain, thereby opening the paperclip conformation. However, the combination of these two epitopes (i) causes the paperclip structure to compress, (ii) brings the N-terminal region closer to the repeat region, and (iii) further strengthens the compression through merging (Jeganathan et al., 2008).

Multiple phosphorylations of these epitopes have been shown to result in the formation of tau filaments and acceleration of tau aggregation. In cases of mild cognitive impairment (MCI), Šimić et al. (2016) observed an increase in phosphorylation at the AT8 and PHF13 epitopes, suggesting that these sites are phosphorylated in parallel. Phosphorylation at the Thr181 site is also increased in MCI, and phosphorylation at this site occurs along with the formation of Aβ plaques approximately 20 years before the onset of AD (Barthélemy et al., 2020).

Since the first case of the disease was reported in 1907, disease-modifying treatments have not been fully available, despite significant progress in research on the pathogenesis of AD, which is recognized as a global public health priority by the World Health Organization (Lane et al., 2018). The “cholinergic hypothesis of age-related cognitive dysfunction” is supported by the selective degeneration of cholinergic neurons in the basal forebrain, and it has been shown that acetylcholinesterase (AChE) inhibitors provide clinical improvement (Chen and Mobley, 2019). Despite the scarcity of treatments to halt or manage AD, FDA-approved AChE inhibitors, including rivastigmine, galantamine, and donepezil, along with memantine, an N-methyl-D-aspartate receptor antagonist, are used to alleviate the symptoms of the condition. Although these medications are considered to enhance cognitive functioning, their efficacy remains contentious (Chen and Mobley, 2019).

Methylene blue (MB), which has recently been advertised on social media as an “elixir of immortality”, is a phenothiazine dye and has received FDA approval for the treatment of methemoglobinemia. MB was used in the malaria epidemic that occurred during World War I and provided beneficial results (Lu et al., 2018). MB is a well-established, potent antioxidant and therapeutic agent that is recommended in the medical field for the treatment of psychotic and neurodegenerative diseases due to its effects on mitochondrial dysfunction (Kayabaşı and Erbaş, 2020). A multitude of studies have been performed on MB to better understand its effect on AD and tau pathology. MB can easily cross the blood–brain barrier (BBB) via its redox mechanism. In vivo studies indicate that MB decreases soluble tau levels and positively affects cognitive functions (O’Leary et al., 2010). The first discovery demonstrating the effects of phenothiazine compounds on tau protein was described by Wischik et al. (1996). In this in vitro research, they defined the tau–tau binding inhibitory constant as 3.4 μM for MB, 98 nM for TH, and 69 nM for TBO, and also showed that reducing the tau–tau binding interaction facilitated the proteolytic degradation of tau aggregates.

The phosphorylation of tau protein is a complex and refined regulatory process that ensures the continuity of neuronal architecture. At the heart of this process, there is a delicate balance between kinases and phosphatases. Among the most prominent kinases, glycogen synthase kinase-3 beta (GSK3β) and cyclin-dependent kinase-5 (Cdk5) phosphorylate tau at multiple serine and threonine residues, weakening its interaction with MTs, thus initiating a pathological transition from structural flexibility to aggregation (Wang et al., 2007). Lee et al. (2003) have shown that GSK3β leads to excessive phosphorylation of tau at Thr 181, Ser 202/Thr 205, Thr 231, and S396/Ser404 in human neuroblastoma cells. GSK3β is not only a key regulator of tau phosphorylation in AD pathogenesis but also has transformative effects on Aβ production and accumulation. This bidirectional interaction creates a profound and amplifying resonance in the molecular architecture of the disease. GSK3β’s promotion of the β- and γ-secretase pathways in APP metabolism increases the production of Aβ peptides, accelerating the neuroinflammatory and synaptotoxic effects of toxic oligomers (Medina and Avila, 2014). Simultaneously, the accumulation of phosphorylated tau impairs Aβ clearance by impairing axonal transport, triggering positive feedback loops that further increase GSK3β activity. This molecular vicious circle results in a synergy that feeds off and potentiates tau and amyloid pathologies. Thus, GSK3β functions not only as an effector in AD but also as a pathogenic amplifier at the core of the tau-amyloid interaction network. Its dual activity represents a molecular chaos that is too profound to be controlled by monotherapies, and thus targeted interventions against GSK3β offer the potential for simultaneous therapeutic benefits at both the tau and amyloid axes (Ferrari et al., 2003). The other central regulator, Cdk5, which determines the structural fate of tau, causes multiple phosphorylations at AD-related epitopes such as Ser396, Ser202, and Thr205, and also phosphorylates Ser235 and Ser404, thus paving the way for GSK3β-mediated phosphorylation (Li et al., 2006). Conversely, protein phosphatase 2A (PP2A), which removes phosphate groups from tau, is the natural antagonist of this phosphorylation process. Normal functioning of PP2A ensures cellular homeostasis by suppressing p-tau levels at many phosphorylated sites. This bidirectional control mechanism ensures not only the functional integrity of tau but also synaptic stability and cellular continuity (Liu et al., 2008).

In our recent research, we characterized the effects of another phenothiazine compound, TBO, on APP processing and tau phosphorylation in N2a/APPSwe cells. In this study, we also investigated AD-related molecular pathways involved in the effects of TBO on tau phosphorylation using the same cell line. It was demonstrated that TBO exhibits an inhibitory effect on tau phosphorylation by reducing levels of Cdk5, GSK3β, Akt, and p-PP2A (inactive form of PP2A) and increasing p-GSK3β (inactive form of GSK3β) and p-Akt levels (Onder et al., 2022). Furthermore, Han et al. (2022) found that MB lowers tau phosphorylation by modulating the Akt/GSK3β signaling pathway in SH-SY5Y neuroblastoma cells in which tau hyperphosphorylation was induced with okadaic acid. MB treatment increased p-Akt and p-GSK3β levels, leading to a reduction in the p-tau Ser396 level. It was also shown that MB reduced tau protein aggregation and prevented cell apoptosis (Han et al., 2022).

The complex nature of AD and the challenge of crossing the BBB have limited the efficacy of treatment approaches. Phenothiazine-structured compounds may serve as a “silver bullet” when rational and straightforward solutions are sought for a complex disease, as they target the cholinergic, amyloid, tau, and molecular pathway hypotheses (O’Leary et al., 2010).

Our present study shows that the highest tested dose, 10 μM TH, significantly decreased the p-tau Ser202/Thr205 level by 73%, p-tau Thr181 level by 60%, p-tau Ser396 level by 21%, and p-tau Ser396/Ser404 level by 55%. Furthermore, the lowest dose tested in our study, 2.5 μM TH, was found to significantly reduce p-tau Ser202/Thr205 levels by 34%, p-tau Thr181 levels by 21%, p-tau Ser396 levels by 29%, and p-tau Ser396/Ser404 levels by 34%. This is the first study to show that TH reduces total tau and p-tau levels at key sites (Ser202/Thr205, Thr181, Ser396, and Ser396/Ser404) involved in NFT formation in an AD-cell model. These results suggest that TH might serve as an intriguing therapeutic agent in tau-associated neurodegeneration.
